# Molecularly imprinted polymer-specific solid-phase extraction for the determination of 4-hydroxy-2(3H)benzoxazolone isolated from *Acanthus ilicifolius* Linnaeus using high-performance liquid chromatography-tandem mass spectrometry

**DOI:** 10.3389/fnut.2022.950044

**Published:** 2022-10-21

**Authors:** Xingbin Ma, Hongling Lin, Yanhong Yong, Xianghong Ju, Youquan Li, Xiaoxi Liu, Zhichao Yu, Cuomu Wujin, Yongxin She, Jiyu Zhang, A. M. Abd El-Aty

**Affiliations:** ^1^Department of Veterinary Medicine, Guangdong Ocean University, Zhanjiang, China; ^2^Zhanjiang Experimental Station, Southern-Subtropical Crop Research Institute, Chinese Academy of Tropical Agricultural Sciences, Zhanjiang, China; ^3^College of Veterinary Medicine, Gansu Agricultural University, Lanzhou, China; ^4^Key Lab of Veterinary Pharmaceutics Development, Ministry of Agriculture/Key Lab of New Animal Drug Project, Gansu Province/Lanzhou Institute of Husbandry Science and Veterinary Pharmaceutical Sciences, Chinese Academy of Agricultural Sciences, Lanzhou, China; ^5^Institute of Veterinary and Animal Husbandry, Tibet Academy of Agricultural and Animal Husbandry Sciences, Lhasa, China; ^6^Institute of Quality Standards and Testing Technology for Agri-Products, Chinese Academy of Agricultural Sciences, Beijing, China; ^7^State Key Laboratory of Biobased Material and Green Papermaking, Qilu University of Technology, Shandong Academy of Sciences, Jinan, China; ^8^Department of Pharmacology, Faculty of Veterinary Medicine, Cairo University, Giza, Egypt; ^9^Department of Medical Pharmacology, Medical Faculty, Ataturk University, Erzurum, Turkey

**Keywords:** 4-hydroxy-2(3H)benzoxazolone, molecularly imprinted polymers, solid-phase extraction, high-performance liquid chromatography, *Acanthus ilicifolius* Linnaeus

## Abstract

The minor constituent found in *Acanthus ilicifolius* Linnaeus, 4-hydroxy-2 (3H) benzoxazolone alkaloid (HBOA), has a range of versatile applications. Herein, a quick and straightforward method for extracting HBOA from *A. ilicifolius* Linnaeus was proposed. HBOA was used as a template, whereas methacrylic acid, ethylene glycol dimethacrylate, and acetonitrile were used as functional monomers, cross-linkers, and porogens, respectively. Molecularly imprinted polymers (MIPs) were synthesized by precipitation polymerization, and their adsorption isotherms, dynamics, and selective binding ability were characterized and analyzed. The results showed that the adsorption amount of the template was 90.18 mg/g. The MIPs were used as solid-phase extraction fillers and actual sample extraction columns, with a linear range of 0–100 μg/L, average recovery of 78.50–101.12%, and a relative standard deviation of 1.20–3.26%. The HBOA concentrations in the roots, stems, and leaves were 1,226, 557, and 205 μg/g, respectively. In addition, MIP–SPE was successfully used in isolating and purifying HBOA from different parts of *A. ilicifolius* Linnaeus, indicating its effectiveness in extracting and determining HBOA in other herbs.

## Introduction

*Acanthus ilicifolius* Linnaeus is a mangrove plant belonging to the genus *Acanthus* in the Acanthaceae family. It grows in the tropical coastal areas of Southeast Asia. Root application has a long history of usage as an herbal remedy, mainly for treating acute and chronic hepatitis, stomach ache, cough, and asthma (1, 2). However, owing to the complex active ingredients of this plant, many problems and critical challenges are encountered in the extraction, isolation, and characterization of active ingredients (3). Hence, low-dose active compounds cannot remarkably exert efficient and sustainable effects for treating diseases.

Current research on *A. ilicifolius* Linnaeus has extensively explored its chemistry and pharmacology. 2-benzoxazolone-type alkaloid is the most isolated active component obtained from *A. ilicifolius* Linnaeus (4). Murty et al. (5) were the first to isolate a 2-benzoxazolone-type alkaloid. They found that it exerts an effect against *Leishmania donovani* and asthma. Furthermore, Tang et al. (6) demonstrated the significant efficacy of benzoxazolone derivatives in treating ALI when the derivatives were induced with lipopolysaccharides. As an effective alkaloid, 4-hydroxy-2(3H)benzoxazolone (HBOA) is vital for ensuring the efficacy of herbs (7, 8). Therefore, establishing a method ensuring the efficient separation and purification of HBOA is of great significance.

Molecular imprinting technologies have been widely used in many fields, such as chromatographic separation (9), solid-phase extraction (10), clinical drug analysis (11), and natural product separation (12). Molecularly imprinted polymers (MIPs) have excellent physical and chemical properties and capability for specialized template molecule identification, separation, purification of the active substance, and efficacy as template molecules. In the specific separation of traditional Chinese medicine, the selection performance of the separation and purification technology is comparable to that of natural antibodies. The technology has unique chemical and mechanical strength, tolerance to acid, alkali, organic solvents, temperature, and pressure, and it is easy to synthesize and store (13). MIPs have high affinity and selectivity, strong resistance to harsh environments, good stability, and long service lives (14). On the other hand, they have poor selective recognition, substantial mass transfer resistance, and slow adsorption dynamics balance. Thus, particular polymers are difficult to obtain.

Since the first isolation and extraction of 2-benzoxazolinone from *A. ilicifolius* in 1984,4-hydroxy-2(3H)benzoxazolone (HBOA) (15), benzoxazolin-2(3H)-one (16), 2H-1,4-benzoxazin-3(4H)-one, and 4-hydroxy-2H-1,4-benzoxazin-3(4H)-one have been isolated through high-speed countercurrent chromatography (17). However, the current application of this extraction technology is still limited concerning isolating active ingredients from herbs.

Currently, SPE mainly includes bonding materials, such as C18 and ion exchange resins. These fillings are not highly selective for targets (18), and while enriching analytes, the fillings also enrich many interfering substances (19), interfering with the final chromatographic analysis. Additionally, the content of active compounds is extremely low in *A. ilicifolius* Linnaeus. Thus, a traditional extraction technology has almost no selective recognition effect, and a high volume of organic solvents is wasted. Finally, obtaining sufficient amounts of HBOA molecules for enrichment and determination remains difficult.

This study prepared and examined novel MIPs based on appropriate template molecules. The optimal ratio of functional monomers to the template and the binding mechanism were investigated using UV spectra. HBOA MIPs are highly selective and can be used in solid-phase SPE packing. Adsorption properties and optimal elution conditions were evaluated for the purification method. Finally, MIP–SPE was developed to extract HBOA from *A. ilicifolius* Linnaeus.

## Experimental

### Materials

Ethanol, toluene, and methanol were secured from Sigma–Aldrich (St. Louis, MO, USA). Methacrylic acid (MAA), diethylene glycol dimethacrylate (EGDMA), and azobisisobutyronitrile (AIBN) were procured from Alfa Aesar (Heysham, UK). All other materials were of analytical reagent grade and acquired from Beijing Chemical Reagent Factory (Beijing, China). *A. ilicifolius* Linnaeus was supplied by Guangdong Huangfu Medicine Co., Ltd. (Zhanjiang, China). Additionally, 4-hydroxy-2(3H)benzoxazolone and matrine (purity > 98%) were obtained from Chengdu Ruiphens Biotechnology Co., Ltd. (Chengdu, China).

### Instruments and chromatography

A Nanodrop 2000C system (Thermo Fisher Scientific, MA, USA) was used to measure the appropriate proportion between the template molecules and a functional monomer. The polymers were also characterized by Fourier transform infrared spectroscopy (FT-IR; PU9800, PANalytical, Almelo, The Netherlands). The selectivity and affinity of an MIP were evaluated using HPLC. The HPLC system consisted of an Agilent Infinity II 1260 (Agilent, Waldbronn, Germany) and a PAD detector running at 274 nm. Chromatographic separation was performed on a C18 column (COSMOSIL C18; 2.1 × 150 mm, 5 μm), purchased from Nacalai Tesque (Kyoto, Japan), held at 30°C. The mobile phase was composed of acetonitrile (A) and 1% (*v/v*) formic acid solution (B; 35:65, *v/v*), and the pH was 5.0. The flow rate was 1 mL min^−1^, with an injection volume of 50 μL. An Agilent series liquid chromatograph coupled with an API 5,000 triple quadrupole mass spectrometer (AB Sciex, USA) was equipped with a turbo ionization spray (ESI) interface.

Scanning electron microscopy (SEM) images were obtained using an S-4800 scanning electron microscope (Hitachi, Tokyo, Japan) with a domain of 5 kV. A Miller-Q ultrapure water filter (Milli hole), 1/10,000 electronic balance (Melter, Switzerland), low-temperature high-speed centrifuge (Thermo Technologies), VSM-3 oscillator (Whirlpool, USA), and Milli-Q ultrapure water machine (Milli-Q, France) were used.

### Preparation of MIPs

HBOA-imprinted polymers were prepared by precipitation polymerization. HBOA and MAA at a mole ratio of 1:4 were dissolved in 5 mL acetonitrile and refrigerated at 4°C for 30 min. Then, 0.6 mmol EGDMA was added as a cross-linker, followed by an initiator (azobisisobutyronitrile, 0.15 mmol) and ACN (50 mL). Nitrogen was applied for 15 min. The solution was placed in a 60°C water bath for 24 h. After polymerization, the polymer particles in the ACN were screened. The solid polymer particles were retained with a 22 μm nylon film. To remove HBOA, polymer particles were extracted through Soxhlet extraction until HBOA was not detected by HPLC. The residues in the MIPs were rinsed with ACN, and NIPs were prepared with the same method without a template. HBOA-MIP was prepared in triplicate until the synthetic amount met the experimental needs.

### Adsorption assays and Scatchard plot analysis

The adsorption properties of MIPs for HBOA and matrine in a mixed standard solution (0.5, 1, 2, 3, 4, 5, 8, 10, and 12 mg/mL) were studied. After achieving adsorption equilibrium, the polymers were separated by MIP and NIP. The active constituents in the supernatant were determined by HPLC. The amount of HBOA adsorbed on MIP\NIP was calculated using the following equation:


$$\begin{array}{l} {\text{q}}_{\text{e}}=({\text{c}}_{0}-{\text{c}}_{\text{e}})\text{ V}/\text{W} \end{array}$$

where *c*_0_ and *c*_*e*_ (mg/mL) are the initial and equilibrium concentrations of compounds in the matrix solution, respectively; *V* (*L*) is the volume of the standard solution; *W*(*g*) is the weight of MIP; and *q*_*e*_ is the static binding capacity.

The Scatchard model calculated the adsorption isotherms. The equation was as follows:


$$\begin{array}{l} {\text{q}}_{\text{e}}/{\text{c}}_{\text{e}}=({\text{q}}_{\text{max}-}{\text{q}}_{\text{e}})/{\text{K}}_{\text{b}} \end{array}$$

where *q*_max_ is the maximum saturated adsorption capacity (mg/g) and *K*_*b*_ is the equilibrium dissociation constant.


$$\begin{array}{l} \text{IF}={\text{q}}_{\text{MIP}}/{\text{q}}_{\text{NIP}} \end{array}$$

IF is the imprinted factor, and *q*_MIP_ and *q*_NIP_ are the imprinted and non-imprinted polymer adsorption to HBOA, respectively (20).

### Selectivity assays

Given that the molecular structure of HBOA is different from that of matrine, the selectivity of matrine was evaluated as a functional object. The selectivity coefficient was the distribution coefficient ratio between HBOA and the other analytes to polymer (21). The selective sites of HBOA and matrine were compared. The relative selectivity coefficient was the selectivity ratio between MIPs and NIPs in a single analyte in a mixed standard solution. The selectivity recognition of MIPs with HBOA was also studied. Ten milligrams (1 g) was added to a mixture solution (1, 5, and 10 mg/mL) containing HBOA and matrine. After adsorption equilibrium, the mixture was subjected to vibration at room temperature for 24 h. Then, the polymers were separated and tested in triplicate. Then, centrifugation was performed (5,000 rpm). The supernatants were analyzed directly with HPLC and measured. Adsorption selectivity was analyzed using *K*_*D*_, α, and β parameters.


$$\begin{array}{l} {\text{ K}}_{\text{D}}={\text{q}}_{\text{e}}/{\text{c}}_{0}\\ \;\alpha ={\text{K}}_{\text{Di}}/{\text{K}}_{\text{Dj}}\\ \beta =\alpha_{\text{MIP}}/\alpha_{\text{NIP}} \end{array}$$

where α is a separation factor; α_*M*_ is the separation factor for the imprinted polymers; *i* and *j* are the template and competing molecules, respectively; α_NIP_ is the separation factor for non-imprinted polymers; and β is a relative separation factor.

### Preparation and procedure optimization of the solid-phase extraction column

A sieve plate was placed at one end of a 10 mL polyethylene solid-phase extraction column, and the thickness faced the polymer. Approximately 10 mg of MIP and NIP were activated with 10 mL of ACN and added to the column. The loading, washing, and elution solvents were optimized for solid-phase extraction columns. The resulting solution was collected and dried at 25°C in the presence of nitrogen. The residue was redissolved in 1 mL ACN, and the resulting solution was filtered with a 22 μm nylon film and analyzed with HPLC. Each test was repeated three times.

The loading solvents investigated were the different volume ratios of ACN-water. Standard solutions, including HBOA and matrine (1 mL, 5 mg/mL), were loaded onto the MIP solid-phase extraction column containing MIP. Washing solvents for *n*-hexane, methylene chloride (CH_2_Cl_2_), carbon tetrachloride (CCl_4_), and ethyl acetate were examined. The eluting solvent had different proportions of acetic acid and methanol (1:9, 2:8, and 3:7, *v/v*).

### Sample analysis

After naturally drying for 2 weeks and crushing with 80 mesh, 100 mg *A. ilicifolius* Linnaeus root, stem, leaf, and seed powder samples were weighed and soaked in 3.0 mL of ammonia solution in 10 mL Erlenmeyer flasks by vortexing for 5 min. Then, 5 mL ACN was added, and ultrasound treatment was performed for 20 min. Centrifugation was conducted at 5,000 rpm for 10 min. The collected supernatants were defatted and enriched. The procedure was repeated five times until the extract became clear. The supernatant was almost evaporated by drying in a stream of nitrogen. Before MISPE, the template (0, 5, 50, and 100 μg/kg) was spiked. The extracts were redissolved and filtered with a 0.22 μm nylon film in 1 mL loading solvent. Under optimum conditions, the above extract was added to the MISPE procedure. The determination method of HBOA in *A. ilicifolius* Linnaeus was validated by HPLC–MS/MS. The chromatographic separation was performed on an analytical column, Agilent Eclipse Plus Phenyl-Hexyl column, 2.1 ID × 100 mm and maintained at 28°C. The mobile phase consisted of 1% formic acid-aqueous solution (A) and 100% acetonitrile B for 15 min. The minimum flow rate was 200 μL min^−1^, and the injection volume was 0.5 mL. The electrospray interface was used in the positive modes; the parent mass was 152.1 (*m/z*), and the daughter mass was 87.0 (*m/z*) as quantitative ions. The collision energy and declustering potential of the HBOA were 25 (eV) and 16 (V), respectively.

## Results and discussion

### Preparation and binding mechanism

**Figure 1:**
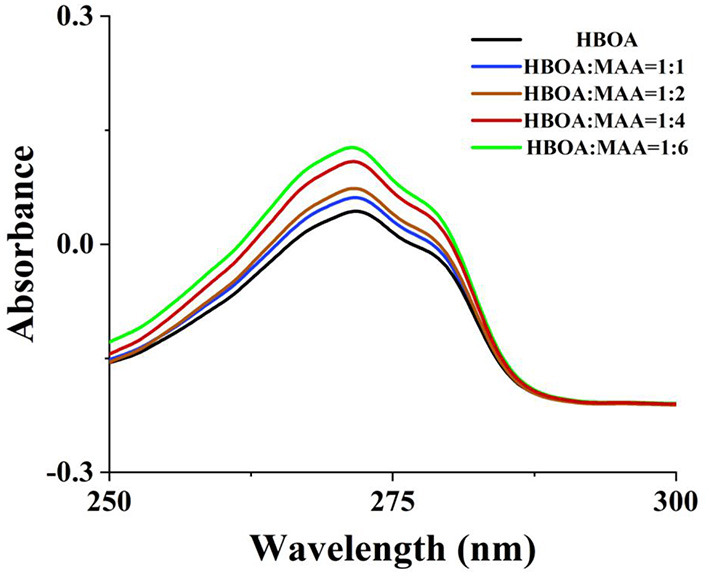
UV absorption spectra of HBOA with the ratio to MAA.

**Figure 2:**
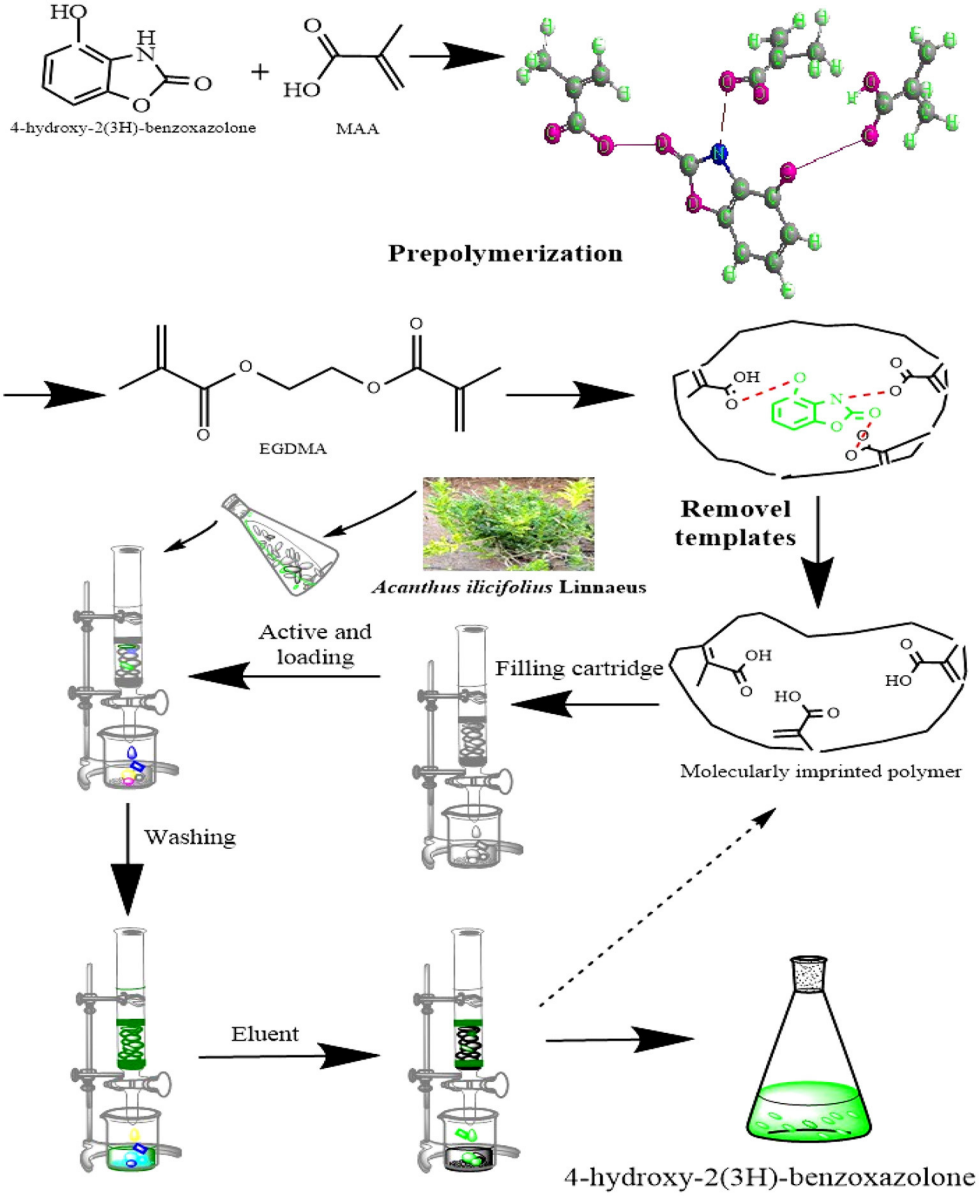
Schematic diagram of the conformation of the associated molecules.

The MIPs prepared through precipitation polymerization have the advantages of an apparent spherical structure and a pure product system. However, the conditions for preparing MIPs through precipitation polymerization are relatively flexible, exhibiting a high sphere formation rate and dependent on the reaction system and types of solvent (22). MIPs were obtained by enabling the binding of the host and object complex (host-gust complex) between HBOA and the MAA hydrogen bond or Π and noncovalent bond (23, 24). The results showed a slightly red band in the UV spectra. There are hydroxyl groups, a tertiary amine, a carbonyl group, and a benzene ring group in the HBOA molecule. MAA was selected as a functional monomer because it favors hydrogen bond or ionic bond interactions in porogens before polymerization (12). Electrostatic interactions are caused by the tertiary amine and carboxyl moieties of MAA, which provide hydrogen and Π-Π stacking interactions from ethylene bonds to HBOA, potentially leading to tight binding between prepolymers. The MAA monomers exhibit not only hydrogen bonding and electrostatic interactions but also stacking of the Π-Π. The molar ratio of ASD to MAA was 1:4 instead of 1:6 (Figure 1). The experiments additionally revealed that the MIPs displayed the highest absorption performance when using HBOA molecules and functional monomers with a ratio of 1:4 under other optimization conditions. When the cross-linker (EGDMA) was added to the complex, the molar ratio between the template and cross-linker was 1:25. Polymerization was triggered by the initiator (AIBN) under heat, and the molar ratio between the template and initiator was 1:4. A polymerization reaction occurred around the imprinted molecule–monomer complex, as shown in Figure 2. In this process, the polymer chain “captured” HBOA molecules and monomer complexes into the stereo-structure of the polymer through free radical polymerization. The imprinted molecules in the polymer were eluted or dissociated by appropriate methods to form binding sites with identified imprinted molecules.

### Characterization

**Figure 3:**
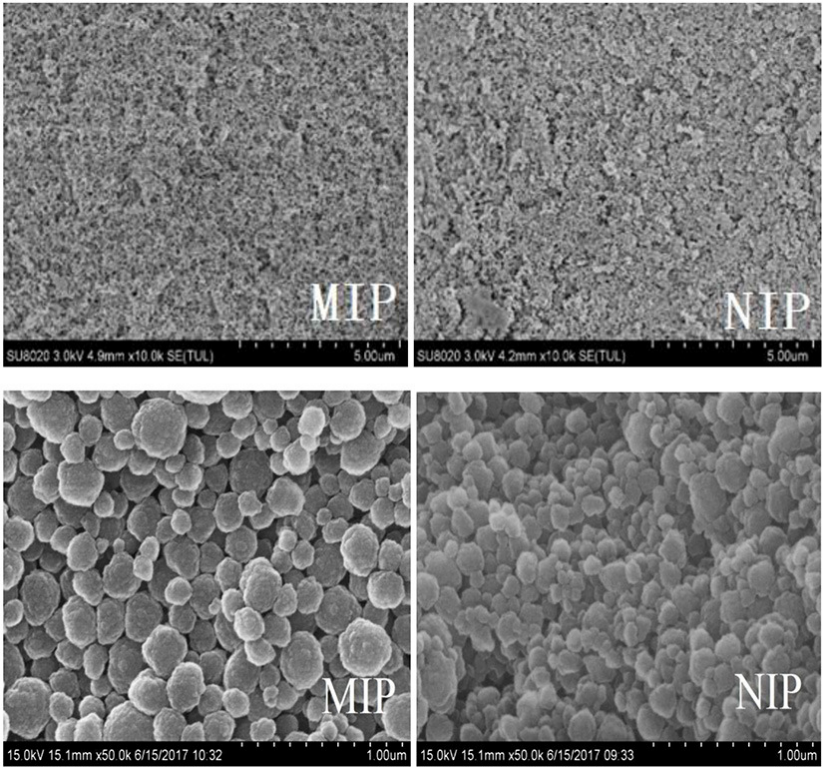
SEM images of NIP and MIP.

**Figure 4:**
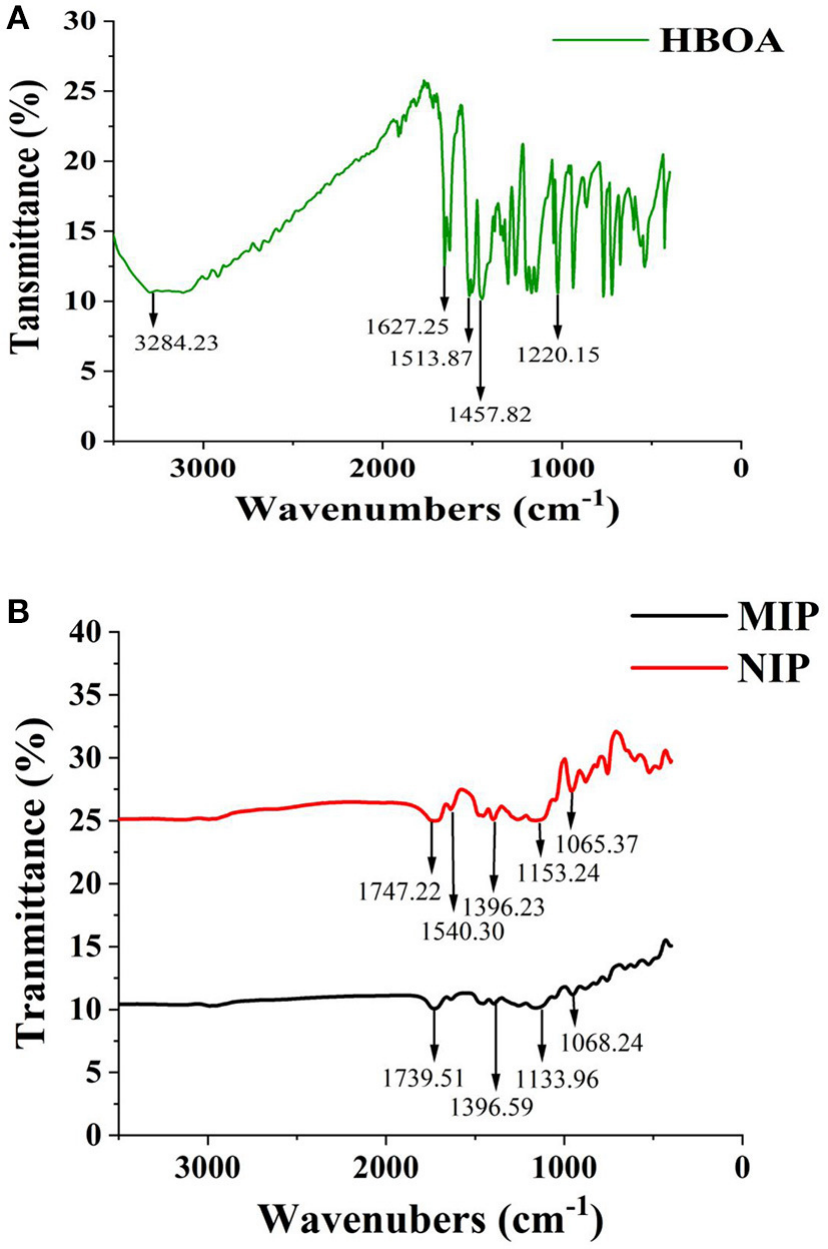
FTIR spectra of the template [HBOA, **(A)**], MIP and NIP **(B)**.

The morphology of MIP and NIP was determined by SEM (Figure 3). The white MIP was filled with holes and had a reticulated surface and a spherical and narrow distribution. The polymerization process increases the active surface area of the MIPs, which improves the polymer extraction capacity. The synthesized MIPs were successfully characterized by the FT-IR spectra of HBOA, MIP, and NIP (Figure 4). The peaks at 3,284 and 1,220 cm^−1^ resulted from –OH and –NH–, respectively. The cyclo-benzene stretching vibration peaks were observed at 1,627, 1,513, and 1,457 cm^−1^, which revealed the presence of HBOA (Figure 4A). After polymerization, the peak at 3,284 cm^−1^ disappeared. Secondary amide (–CONH–) stretching vibration peaks at 1,540 cm^−1^. A comparison of the NIP revealed that the template binds to the MIP. This shift from 1,747 to 1,739 cm^−1^ suggests that the free C=O in the template may be related to the hydrogen bond formed (25). Stretching vibration peaks of –C–O– were observed at 1,396, 1,153, and 1,065 cm^−1^, which could be from EGDMA (Figure 4B). When the functional polymer monomer was the same and the template molecular binding group was the same, the role of the imprinted hole became a key factor for determining specific binding. In fact, the holes were recognized as a molecular sieve. When small compounds were used as template molecules, most of the large compounds could not enter the imprinted hole. These large compounds were used as template molecules. Smaller compounds could enter the imprinted hole more readily than template molecules and then occupy the imprinted hole by acting with the functional base.

### Isothermal adsorption and desorption rate

**Figure 5:**
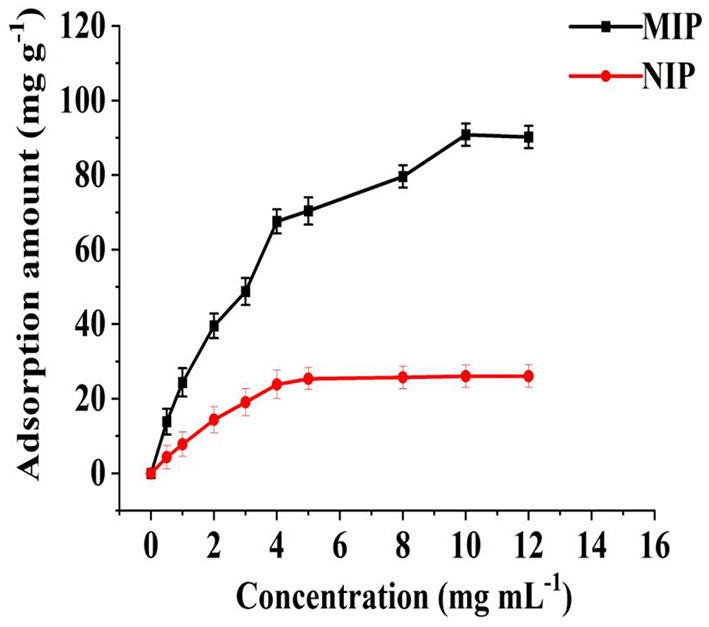
Adsorption isotherm diagram of NIP and MIP for HBOA.

The adsorption of MIP and NIP to different concentrations of HBOA was determined, and isothermal adsorption lines were drawn. As shown in Figure 5, when the HBOA concentration reached 10 mg/L, the specific recognition sites on the MIP were occupied entirely, and the adsorption amount to HBOA reached saturation. Moreover, the adsorption-desorption processes reached dynamic equilibrium. The adsorption amount of the MIP was 90.83 mg/g. Given that NIPs do not have specific recognition sites, HBOA was not specifically adsorbed, so the capture capacity of HBOA was poor, and the HBOA adsorption amount was significantly lower than that of the MIP. The maximum adsorption amount of NIP was 21.58 mg/g.

**Figure 6:**
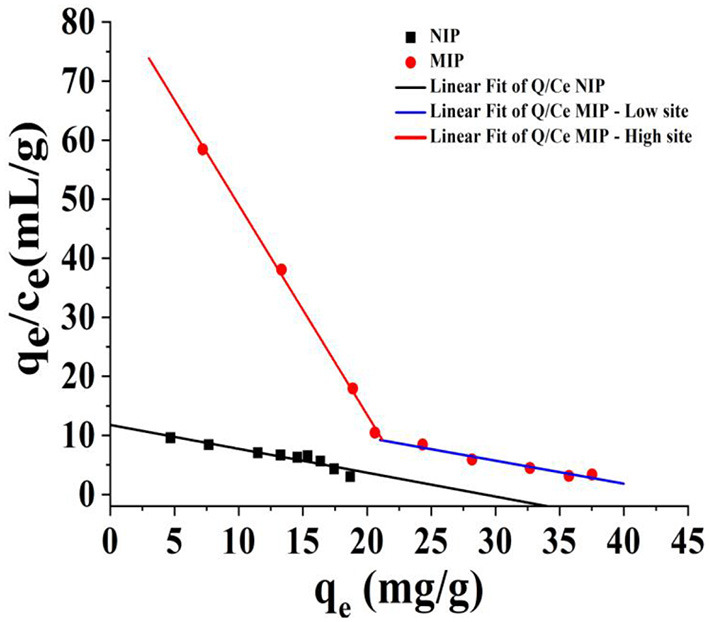
Scatchard plot analysis of MIP for HBOA.

To evaluate the recognition performance of the MIP, the isothermal adsorption data of HBOA were used with MIP in the Scatchard analysis. Mapping *q*_*e*_ with *q*_*e*_/*c*_*e*_, Linear fit of Q/Ce—MIP—high site (*q*_*e*_/*c*_*e*_ = 84.55 – 3.55*q*_*e*_, *R*^2^ = 1.0), Linear fit of Q/Ce—MIP—low site (*q*_*e*_/*c*_*e*_ = 17.41 – 0.40*q*_*e*_, *R*^2^ = 0.99), Linear fit of Q/Ce—NIP—site (*q*_*e*_/*c*_*e*_ = 11.76 – 0.32*q*_*e*_, *R*^2^ = 0.93), two better linear lines were found, which denoted that two classes of binding sites were found in the synthetic polymers (Figure 6). According to the slope and intercept (26), the equilibrium dissociation constant of HBOA for the polymer to the high-affinity binding site (*K*_*b*_) of MIP was 0.28 mg/L, the theoretical maximum binding amount (*q*_max1_) was 84.55 mg/g, and the equilibrium dissociation constant to the low-affinity binding site (*K*_*b*_) of MIP was 2.5 mg/L. The theoretical maximum binding amount (*q*_max2_) was 17.41 mg/g. The equilibrium dissociation constant of HBOA for the polymer to the binding site (*K*_*b*_) of NIP was 3.13 mg/L, and the theoretical maximum binding amount (*q*_max2_) was 11.76 mg/kg. Binding site formation was associated with the interaction force of the template, functional monomer, and high-affinity binding sites. High-affinity sites may have played a central role in the static site (27). This finding indicates that the MIP compared with NIP has good affinity and adsorption properties.

**Figure 7:**
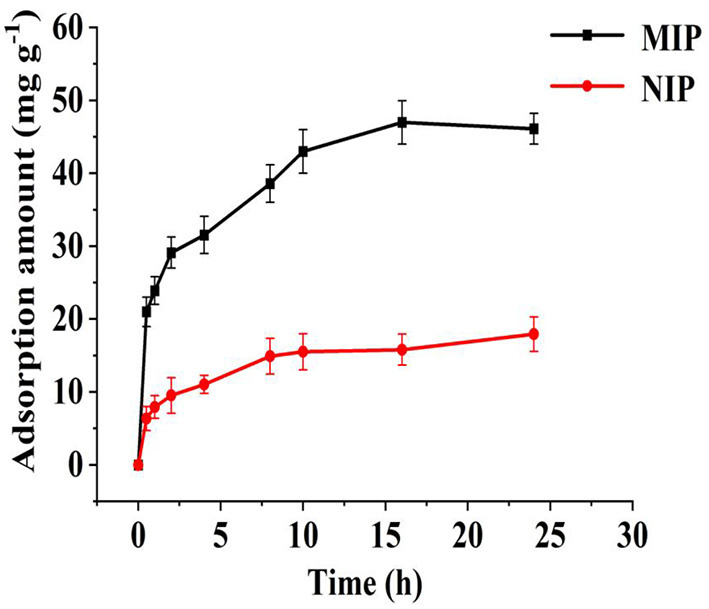
Static adsorption curve of MIP for HBOA.

As shown in Figure 7, the specific bonding point on MIP was not occupied at 0–8 h, and the adsorption amount increased rapidly with time (from 10 to 24 h). The number of specific recognition sites on the MIPs gradually decreased, and the adsorption amount increased and tended to be stationary. After 16 h, the polymer reached the maximum adsorption amount, and the polymer adsorption-desorption processes reached equilibrium. On the NIPs, at 0–5 h, the adsorption amount increased rapidly over time. After 5 h, the NIP adsorption-desorption processes reached equilibrium, and almost no adsorption was observed. Imprinting factors were used to evaluate the printing effect of HBOA in the imprinted polymers. The imprinting of the factor was maintained at ~1.78, indicating that HBOA was successfully imprinted in the polymer. An HBOA cavity was left on the polymer when eluted with an elution agent. When adsorbed with HBOA again, HBOA matched the hole. This effect denoted that the prepared MIP has a selective recognition ability for HBOA, thus enhancing the MIP adsorption for HBOA, whereas NIP does not have this function.

### Selectivity

The specific binding of functional bases to template molecules determines the selectivity of an MIP. Selectivity and the number of functional groups bound to the template molecules increased with the number of functional groups (28). To investigate the selective recognition ability of MIPs, a matrine was selected as a distractor for a selective adsorption assay. MIPs have good adsorption properties. Therefore, this experiment examined the selectivity of MIPs for each substrate molecule. The results are shown in Table 1. The MIP adsorption to matrine was very low and barely detectable. The distribution coefficient of MIP to HBOA was significantly higher than that of matrine, indicating the selective adsorption of HBOA for MIP. The *K*_*D*_ (0.146) of HBOA was greater than that of matrine, and the adsorption capacity of MIP was high. The α_*M*_ value of matrine was larger than that of HBOA, indicating that the MIP had an extremely strong selection.

**Table 1:** The selectivity of MIP and NIP for HBOA compared to matrine.

**Sorbents**	**MIP**		**NIP**		**β**
	***K_*D*_* (mL/g)**	**α_MIP_**	***K_*D*_* (mL/g)**	**α_NIP_**	
HBOA	0.146	1	0.0876	1	1
Matrine	0.023	6.33	0.011	7.96	0.80

### Method verification

According to the IUPAC recommendation and previous reports (29–31), the developed MIP as a sample pretreatment was evaluated in terms of linear range, correlation coefficients (*R*^2^), limits of detection (LODs) and quantification (LOQs) for the determination of HBOA and matrine using HPLC–MS/MS. The calibration curve was good in the range of 5–1,000 μg/L for HBOA and matrine at the five concentration levels. The correlation coefficient was 0.9990 for HBOA by determining the standard solution with the MISPE protocol. The limits of detection and quantification were 1.56 and 2.85 μg/L, respectively (Table 2).

**Table 2:** Linear range, linear regression equation, correlation coefficients (*R*^2^), detection limits of HBOA and matrine using HPLC–MS/MS.

**Analytes**	**Linearity (μg/L)**	**Regression equation**	***R^2^***	**LOD (μg/L) (*n* = 5)**	**LOQ (μg/L) (*n* = 5)**
HBOA	5–1,000	*Y* = 356*x* + 0.0025	0.9990	1.56	2.85
Matrine	5–1,000	*Y* = 271*x* + 0.0084	0.9987	2.23	4.30

### Optimization of solid-phase extraction

**Figure 8:**
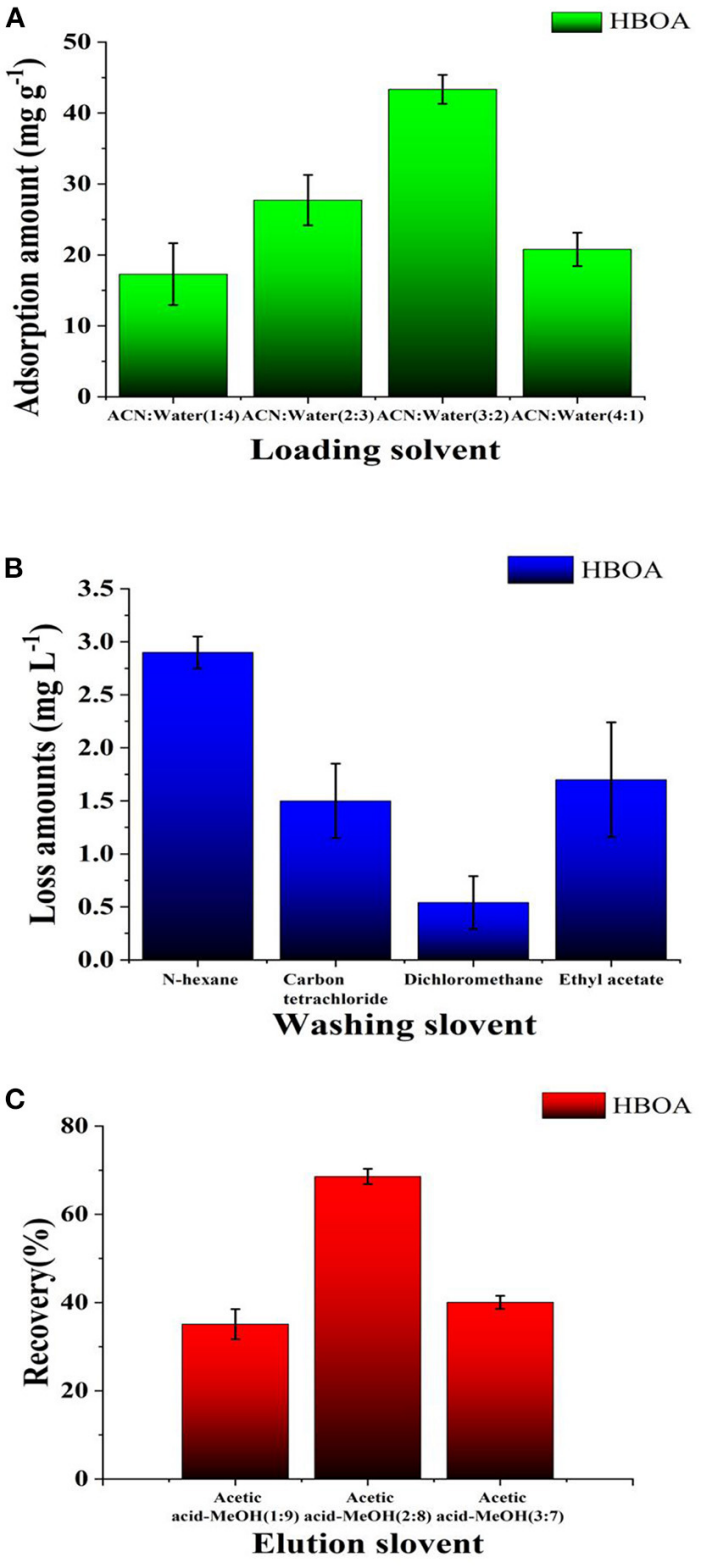
The loading ratio **(A)**, loss ratio **(B)**, and recoveries **(C)** obtained from the MISPE cartridges in the optimized procedure.

Simple MIP–SPE equipment can separate and concentrate the solid-phase extraction process into a one-step, simple, efficient, and flexible method for current sample preprocessing (32). In the present study, based on the solubility of HBOA, different ACN-water ratios were tested to obtain crude extracts of *A. ilicifolius* Linnaeus. As the CAN volume increased, HBOA was extracted in the solid-phase extraction cartridge, and the retention rate gradually decreased. HBOA is insoluble in water, so the crude extract was soluble in 60% ACN aqueous solution (Figure 8A). When the ACN volume was >60%, HBOA retention in the solid-phase extraction cartridge was low. Impurities were washed with an aqueous solution containing 60% ACN fraction as a loading solvent.

The washing step aimed to weaken or break the interactions between impurities and polymers (33). The adsorption force of the selected impurities in the micropores on the microsphere surface was relatively smaller than that of HBOA and was easy to wash. Different weakly polar solvents were used as cleaners in this test. The retention of HBOA decreased with increasing solvent polarity and was evident with CCl_4_. To wash out more impurities, CH_2_Cl_2_ was used as a washing solvent (Figure 8B). Showing little HBOA penetration, the cartridge was not without restriction. HBOA retention in the cartridge decreased as detergent usage increased. When the HBOA dosage was <12.0 mL, CH_2_Cl_2_ penetration was low and had no significant change. HBOA was heavily washed out when the washing dosage was >12.0 mL. To remove impurities and reduce HBOA leakage, CH_2_Cl_2_ (12.0 mL) was selected as a detergent (1.2 times the column bed volume).

The elution was selected according to the solvent polarity (34), and rapid recovery rates of HBOA within 9.0 mL with methanol consumption were observed. When the acetate–methanol dosage was >10.0 mL, the recovery rate remained essentially unchanged, indicating that HBOA was almost completely washed down at 10.0 mL. Owing to cost and recovery issues, acetate-methanol (10.0 mL*, 2:8, v/v*) was selected as an eluting solvent (Figure 8C), which was used for removing the template (HBOA).

### Validation of the MISPE protocol in real samples

**Figure 9:**
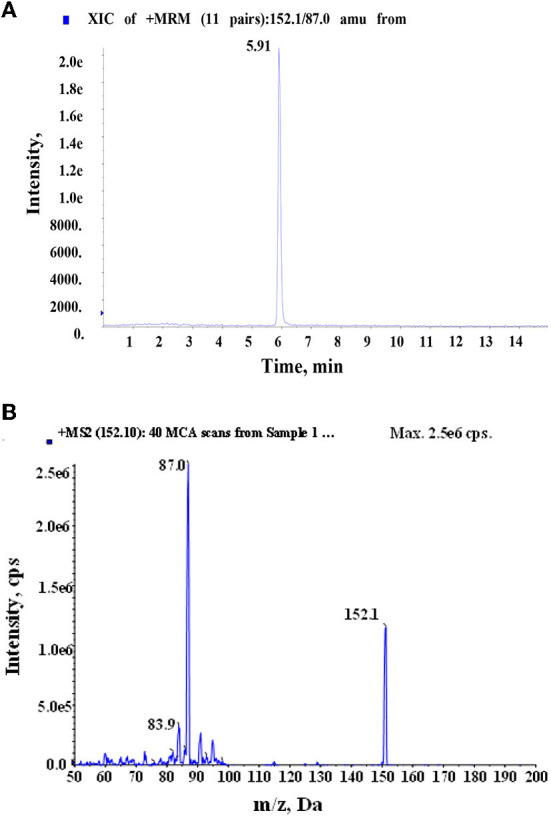
Total ion chromatogram **(A)** of HBOA in *A. ilicifolius Linnaeus* and daughter mass **(B)** of HBOA after MISPE extraction and determination by HPLC–MS/MS.

Approximately 0.1 g of roots, stems, and leaves of *A. ilicifolius* Linnaeus were analyzed through solid-phase extraction and determination coupled with HPLC–MS/MS (35). The detected amounts of HBOA in the roots, stems, and leaves were 1,226, 557, and 205 μg/g, respectively. *A. ilicifolius* Linnaeus is one of the medicinal mangrove plants used as a traditional drug in China and other countries in the Asia-Pacific region (1, 36). Meanwhile, 2-benzoxazolone alkaloids are important active components (7). In this protocol, the accuracy of the proposed method was assessed by adding HBOA at four concentrations (0, 5, 50, and 100 μg/kg). The recovery rate ranged from 78.50 to 101.12%, and the RSD ranged from 1.20 to 3.26% (Table 3). After elution, HBOA was completely eluted and showed good enrichment performance (Figure 9). The successful application of MISPE to HBOA for extraction and determination revealed 1,820, 775, and 242 μg/g in the roots, stems, and leaves, respectively.

**Table 3:** Accuracy of the MISPE-HPLC–MS/MS method for the determination of HBOA in real samples.

**Analytes**			**HBOA**	
***A. ilicifolius* Linnaeus**	**Real content of HBOA (μg/g)**	**Fortified content (μg/kg)**	**Recoveries (%) (*n* = 5)**	**Interday precision (RSD, %) intraday (*n* = 5)**
Leaves	205	0	82.25	1.34
		5	78.50	1.46
		50	80.47	1.20
		100	82.15	2.30
Stems	557	0	81.31	3.26
		5	79.46	2.50
		50	94.24	1.62
		100	97.17	1.26
Roots	1,226	0	82.22	1.25
		5	90.53	1.27
		50	101.12	1.32
		100	99.28	1.56

## Conclusions

HBOA MIPs successfully synthesized in this study exhibited mesoporous molecular sieve surfaces and displayed good specific selectivity. Extraction materials were used as adsorbents for solid-phase extraction. The HBOA content in the different parts of *A. ilicifolius* Linnaeus was analyzed using the proposed MISPE–HPLC–MS/MS method. The concentrations of HBOA were verified in the whole herb. This method can be helpful as a tool for detecting and quantifying HBOA in a variety of herbs.

## Data availability statement

The original contributions presented in the study are included in the article/supplementary material, further inquiries can be directed to the corresponding authors.

## Author contributions

XM and HL wrote the first draft of the manuscript. All authors contributed to the study conception and design, data acquisition, analysis and interpretation of data, critical revision of the manuscript for important intellectual content, and read and approved the final manuscript.

## Funding

This study was supported by the Natural Science Foundation of Guangdong Province, China (No. 2022A1515010576); the Project of Guangdong International, Hong Kong, Macao and Taiwan high-end talent exchange program (Nos. K21425, 010306052102), and the Program for Scientific Research of Guangdong Ocean University (Nos. R20063 and 040401052201).

## Conflict of interest

The authors declare that the research was conducted in the absence of any commercial or financial relationships that could be construed as a potential conflict of interest.

## Publisher's note

All claims expressed in this article are solely those of the authors and do not necessarily represent those of their affiliated organizations, or those of the publisher, the editors and the reviewers. Any product that may be evaluated in this article, or claim that may be made by its manufacturer, is not guaranteed or endorsed by the publisher.
